# Molecular Regulation of Lipogenesis, Adipogenesis and Fat Deposition in Chicken

**DOI:** 10.3390/genes12030414

**Published:** 2021-03-13

**Authors:** Sara Nematbakhsh, Chong Pei Pei, Jinap Selamat, Noordiana Nordin, Lokman Hakim Idris, Ahmad Faizal Abdull Razis

**Affiliations:** 1 Laboratory of Food Safety and Food Integrity, Institute of Tropical Agriculture and Food Security, Universiti Putra Malaysia (UPM), Serdang 43400, Selangor, Malaysia; saranematbakhsh@gmail.com (S.N.); jinap@upm.edu.my (J.S.); noordiana@upm.edu.my (N.N.); 2 Faculty of Health and Medical Sciences, School of Biosciences, Taylor’s University, Subang Jaya 47500, Selangor, Malaysia; peipei.chong@taylors.edu.my; 3 Department of Food Science, Faculty of Food Science and Technology, Universiti Putra Malaysia (UPM), Serdang 43400, Selangor, Malaysia; 4 Department of Veterinary Preclinical Sciences, Faculty of Veterinary Medicine, Universiti Putra Malaysia (UPM), Serdang 43400, Selangor, Malaysia; hakim_idris@upm.edu.my; 5 Natural Medicines and Products Research Laboratory, Institute of Bioscience, Universiti Putra Malaysia (UPM), Serdang 43400, Selangor, Malaysia

**Keywords:** transcriptional regulation, post-transcriptional regulation, adipogenesis, lipogenesis, fat deposition, meat quality, chicken

## Abstract

In the poultry industry, excessive fat deposition is considered an undesirable factor, affecting feed efficiency, meat production cost, meat quality, and consumer’s health. Efforts to reduce fat deposition in economically important animals, such as chicken, can be made through different strategies; including genetic selection, feeding strategies, housing, and environmental strategies, as well as hormone supplementation. Recent investigations at the molecular level have revealed the significant role of the transcriptional and post-transcriptional regulatory networks and their interaction on modulating fat metabolism in chickens. At the transcriptional level, different transcription factors are known to regulate the expression of lipogenic and adipogenic genes through various signaling pathways, affecting chicken fat metabolism. Alternatively, at the post-transcriptional level, the regulatory mechanism of microRNAs (miRNAs) on lipid metabolism and deposition has added a promising dimension to understand the structural and functional regulatory mechanism of lipid metabolism in chicken. Therefore, this review focuses on the progress made in unraveling the molecular function of genes, transcription factors, and more notably significant miRNAs responsible for regulating adipogenesis, lipogenesis, and fat deposition in chicken. Moreover, a better understanding of the molecular regulation of lipid metabolism will give researchers novel insights to use functional molecular markers, such as miRNAs, for selection against excessive fat deposition to improve chicken production efficiency and meat quality.

## 1. Introduction

Chicken, as an economically important agricultural avian species, is currently considered an ideal experimental animal model for biomedical research fields, including the study of the biological mechanisms and genetic regulation of fat metabolism [1]. Besides, sequence alignment of the chicken and human genome indicates that there is a fundamental resemblance between chicken and human genomes, and almost 60 % of chicken genes are highly similar to human genes [2]. Therefore, investigating the mechanism of lipogenesis, adipogenesis, and fat deposition in chicken can produce useful comparative information for human obesity studies and biomedical research and also can be beneficial for the poultry industry to improve the genetic manipulation to reduce abdominal fat deposition in chickens [2,3]. Since abdominal, skin, and intramuscular fat are three major determinants of chicken carcass quality [4], information regarding the regulatory mechanism of transcription factors, miRNAs, fat metabolism-related genes, and their interaction, which control fat metabolism and deposition, might lead to improvement of meat quality in chicken.

De novo fatty acid synthesis in other animals, such as pigs, mainly happens in the adipose tissue, while lipid biosynthesis in chicken mostly (90%) occurs in the liver [5,6]. Fat metabolism is a polygenic trait, which is controlled by several genes and regulatory factors through different signaling pathways. Among factors affecting chicken meat quality, the level of lipogenesis in chicken liver and fat deposition in the muscle are among the most important factors, which determined the quality and health of the meat [7]. On the other hand, chicken as an affordable protein-based food source has undergone intensive selection for rapid growth and meat yield resulting in significant fat deposition, specifically in the abdominal cavity. This excessive fat deposition is the main problem of the chicken industry because it has a promising negative impact on feed efficiency and meat quality and can cause great economic loss [8]. Therefore, studying the molecular regulatory mechanisms of lipogenesis, adipogenesis, and fat deposition at a transcriptional and post-transcriptional regulatory level, will enable us to understand how genes and regulatory factors, including transcription factors and miRNAs, and their interaction affect fat metabolism and carcass quality in chickens [5,9].

Regulation at the transcriptional level involves proteins known as transcription factors that bind specifically to the promoter region of their target genes and control their expression in different metabolic pathways [10]. For instance, in transcriptional regulation of hepatic lipogenesis, the promoter region of lipogenic genes, such as fatty acid synthase (FASN), is targeted by transcriptional factor named sterol regulatory element-binding protein 1 (SREBP 1) through fatty acid and fat synthesis metabolic pathway, which has a significant role in the regulation of fat metabolism in the liver of chicken [11].

It is well understood that a significant part of the eukaryotic genome is transcribed to non-protein-coding RNAs (ncRNAs), which have different sizes ranging from 20 nucleotides to 100 kb and have vital roles in regulating many biological processes, including lipid metabolism. Among these ncRNAs, microRNAs have attracted attention [12], and are progressively recognized as critical regulators of cholesterol and fatty acid homeostasis, lipid metabolism [3], adipogenesis [13,14], and fat deposition [6] through regulating the expression of target genes at the post-transcriptional level. Since miRNAs, as endogenous post-transcription regulatory factors, regulate the expression of various genes involved in lipid metabolism in chicken, constructing miRNA-mRNA interaction networks analysis enable researchers to indicate miRNAs and their target genes, which have an important role in the regulation of fat metabolism [15]. For example, it is revealed that the expression of gga-miR-22-3p is negatively correlated with the expression of ELOVL fatty acid elongase 6 gene (ELOVL6), which is responsible for the last stage of endogenous saturated fatty acid synthesis in de novo lipogenesis in the chicken liver [16]. Likewise, various studies have focused on the study of the differentially expressed miRNAs in abdominal adipose tissue [12,17], intramuscular fat [14], and liver [18] at different ages, developmental stages, and chicken breeds. Furthermore, integrated studies of significant differentially expressed (SDE) miRNAs and Kyoto Encyclopedia of Genes and Genomes (KEGG) pathway analysis enable scientists to identify the significant enriched metabolic pathways related to fat metabolism in chicken to evaluate the pattern of regulation mechanism of adipogenesis, lipogenesis and fat deposition in different chicken breeds [15].

Due to the importance of fat deposition as a factor influencing feed efficiency, the nutritional value of carcass parts, and consequently, the commercial value of chicken [19], the objective of the current review is to provide new insight for deciphering the molecular mechanism underlying fat metabolism and deposition in chicken at the transcriptional and post-transcriptional level. Besides, a brief overview regarding the biochemistry of chicken lipogenesis, adipogenesis, and fat deposition is provided to better understanding the molecular regulation mechanism of fat metabolism at the transcriptional and post-transcriptional level.

## 2. Overview of Lipid Metabolism in Chicken

**Figure 1:**
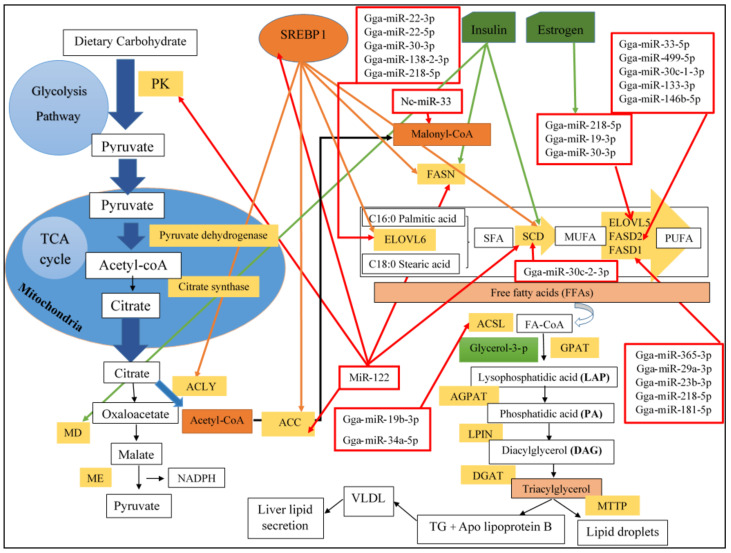
Schematic representation of de novo lipogenesis in chicken hepatocyte and its regulation at the transcriptional and post-transcriptional level. Red, orange, and green lines indicated the targets of various miRNAs, sterol response element-binding protein (SREBP1) as a transcription factor, and hormones in the chicken hepatocyte, respectively. PK, pyruvate kinase enzyme; TCA, tricarboxylic acid cycle; ACLY, ATP-citrate lyase; ACC, acetyl-CoA carboxylase; MD, malate dehydrogenase; NADPH, nicotinamide adenine dinucleotide phosphate; ME, malic enzyme; FASN, fatty acid synthase; ELOVL6, ELOVL fatty acid elongase 6; SCD, stearoyl-CoA desaturase; FADS, fatty acid desaturase; FA-CoA, fatty acid-acyl-CoA; ACSL, long-chain acyl-CoA synthetases; G3P, glycerol-3-phosphate; GPAT, glycerophosphate acyltransferase; AGPAT, acylglycerophosphate acyltransferase; LPIN, lipid phosphate phosphohydrolase; DGAT, diacylglycerol acyltransferase; MTTP, microsomal triglyceride transfer protein; VLDL, very-low-density lipoprotein; SFA, saturated fatty acid; USFA, unsaturated fatty acids; MUFA, monounsaturated fatty acids; PUFA, polyunsaturated fatty acids; FFA, free fatty acids.

In chicken, lipids are mostly represented by triacylglycerol (TG) and are firstly synthesized in hepatocytes and then stored in adipocytes. In comparison with mammals, there are some differences regarding lipid metabolism in chicken, such as the transportation of dietary lipids to the liver, hepatic lipogenesis, and the presence of unique lipoproteins in the blood. It has been evaluated that the main proportion (70%) of fatty acid synthesis in chicken occurs in the hepatocytes through the process named lipogenesis (Figure 1), and only 5% happens in the chicken adipose tissue. However, the remaining (25%) of fatty acids come from the diet [16,20]. After fatty acid synthesis, lipids in the form of very low-density lipoprotein (VLDL) are secreted by the liver and transported through blood circulation to target tissues (e.g., adipose tissue), where the lipoprotein for immediate use or deposition, are hydrolyzed by lipoprotein lipase (LPL) [6]. The fatty acids released from VLDL can penetrate adipocytes, which inside adipocytes resynthesized into triacylglycerol and deposited [20]. In addition, lipid metabolism in chicken is regulated by several hormones, such as insulin and estrogen, as well as key enzymes associated with lipid metabolism [20] (Figure 1). For example, the main enzymes involved in lipogenesis are malate dehydrogenase (MD) and fatty acid synthase (FASN) [21]. Moreover, adipose tissue development in a domesticated animal, such as chicken, depends on species, sex, age, management condition, and the fat depots. Diversity in fat depots is noteworthy in animal production, since it can affect economic and human health [22]. It should be noted that there is a balance among different sources of lipid deposition in chicken, including dietary absorbed fat, lipogenesis, and fat catabolism [23].

### 2.1. De Novo Lipogenesis in Chicken Liver

In birds, including chickens, the liver is the main place for lipogenesis, which provides lipids for usage to all tissues and to the liver itself [22]. The de novo lipogenesis in the hepatic cells of chicken is comprised of de novo fatty acid synthesis, TG synthesis, and the export of synthesized TG from the liver through the bloodstream to peripheral tissues [24] (Figure 1). Chicken hepatic cells can synthesize saturated fatty acids from non-lipid substrates (carbohydrates from diet) and then oxidizing them to mono and polyunsaturated fatty acids [25]. Although TG is the main product of de novo hepatic lipogenesis, the liver also is the main site for phospholipid and cholesterol synthesis [20]. Since poultry feed formulation usually contains less than 50 g/kg fat, which is considered as low-fat feed, the liver plays a dominant role in de novo lipogenesis and conversion of glucose to TG through different pathways [25]. The upstream pathway of lipogenesis in chicken hepatocytes is the glycolysis pathway (cytoplasmic pathway) which is activated by the dietary carbohydrate intake through activation of hexokinase domain containing 1 (HKDC1) gene, encoding for the hexokinase isoform that phosphorylates glucose into glucose 6-phosphate. Subsequently, at the last step of the glycolysis pathway, pyruvate is synthesis in the cytoplasm of chicken hepatocytes by mediating the PK enzyme. Then, the mitochondrial conversion of pyruvate into acetyl-CoA occurs by activation of pyruvate dehydrogenase E1 subunit β (PDHB) and dihydrolipoamide acetyltransferase (DLAT) genes that encode for pyruvate dehydrogenase complex (a complex of three enzymes) [24]. Afterward, the synthesized acetyl-CoA, the main component of lipogenesis [26], participates in the TCA cycle as a citrate source. In the TCA cycle, oxaloacetate, along with acetyl-CoA by mediating citrate synthase, transforms to the citrate in the hepatocyte mitochondria. Later, in the first step of the cellular fatty acid synthesis, acetyl-CoA enters the cytoplasm in the form of citrate and converts to acetyl-CoA and oxaloacetate by ACLY enzyme. Besides, the oxaloacetate is converted to malate employing cytoplasmic MD, which is encoded by the malate dehydrogenase 1 (MDH1) gene [24]. Cytoplasmic acetyl-CoA that is essential for fatty acid biosynthesis should be in its activated form, malonyl—CoA, which this activation is catalyzed by ACC enzyme. Afterward, the conversion of malonyl-CoA to fatty acid is catalyzed by the FASN enzyme [24]. In chicken, the first products of de novo fatty acid synthesis are SFAs, including palmitic acid (C16:0) [26], which then genes, such as ELOVL6, can catalyze the elongation of palmitic acid (C16:0) to create stearic acid (C18:0). Subsequently, through initial desaturation of saturated fatty acids to monounsaturated fatty acids, SCD catalyzes the desaturation of palmitic and stearic acid to palmitoleic acid (C16:1) and oleic acid (C18:1), respectively [6,27]. Further fatty acids elongation and desaturation are catalyzed via enzymes encoded by ELOVLs, as well as FADS1 and FADS2 [15] (Figure 1), which ELOVLs are essential for the rate-limiting step in the elongation cycle during the synthesis of long- and very-long-chain fatty acids (LCFA, VLCFA) [23]. For instance, ELOVL5 can use C18:1 as a substrate to synthesize linolenic acid (C18:3) and arachidonic acid (C20:4) [15]. On the other hand, FADS2, as one of the key limiting enzymes in the lipid metabolic pathway, converts linoleic acid (C18:2) and α-linolenic acid (C18:3) to PUFAs, including docosapentaenoic acid (DPA) (C22:5) and docosahexaenoic acid (DHA) (C22:6). It is indicated that PUFA can act as a regulator for lipid metabolism, and their composition in poultry adipose tissue can directly affect the meat flavor and quality [28]. Finally, after de novo fatty acid synthesis in the liver, fatty acids incorporate into TG for storage in the chicken liver, as well as other tissues [24] (Figure 1).

#### 2.1.1. Triglycerides Synthesis in Chicken Liver

Fatty acids are stored for later use as TG in all cells, but initially in adipocytes of adipose tissue. TG can be synthesized from three sources; circulating FFAs derived from the diet, lipolysis, and de novo TG biosynthesis in the chicken liver [29]. The major pathways involved in the de novo TG biosynthesis in the liver of chicken are the glycerol-3-phosphate (G3P) pathway and the monoacylglycerol pathway [30,31]. G3P pathway, which is comprised of four stages, is the main pathway for most TG synthesis in poultry (Figure 1) [32]. Initially, the activation of synthesized fatty acids into Acyl-CoA is catalyzed by the ACSL enzyme [12]. Subsequently, the first step of the G3P pathway is a conversion of G3P and Acyl-CoA to form 1-acylglycerol-3-phosphate (lysophosphatidic acid, LPA), which is catalyzed with the GPAT gene family. On the second step of this pathway, the AGPAT gene family encodes the enzyme catalyzing the conversion of LPA to phosphatidic acid (PA). Afterward, the LPIN gene family dephosphorylate the PA and form diacylglycerol (DAG) on the third step of the pathway. Finally, DAG is converted to TG by mediating the DGAT1 and DGAT2 genes. It should be noted that DGAT1 has not been found in the chicken genome; however, studies have indicated that chicken sterol acyltransferase 1 (SOGAT1) can be classified into DGAT gene family because it has DGAT1 enzyme activity [30,31,32] (Figure 1).

#### 2.1.2. Lipoprotein Assembly and Secretion

Same as other species, in chicken, the fatty acids synthesized in the liver are significantly incorporated as TG into VLDL and secreted into the plasma [20]. In this regard, the MTTP gene cooperates in the lipoprotein assembly to form low-density lipoprotein [29] (Figure 1). Main lipoprotein particles that are synthesized and secreted by the liver are VLDL, intermediate-density lipoproteins (IDL), low-density lipoprotein (LDL), and high-density lipoproteins (HDL) [20]. Moreover, in different chicken breeds, long-chain fatty acids are gathered into a package named “portomicrons” that are straight delivered into portal blood from the small intestine, which are another type of lipoproteins [31]. In the laying hens, another type of lipoprotein, named vitellogenin, is synthesized by the liver under the control of estrogen during the egg production phase and transferred directly to the ovaries to participate in egg yolk formation [25].

#### 2.1.3. Lipoprotein Degradation and Fat Deposition

**Figure 2:**
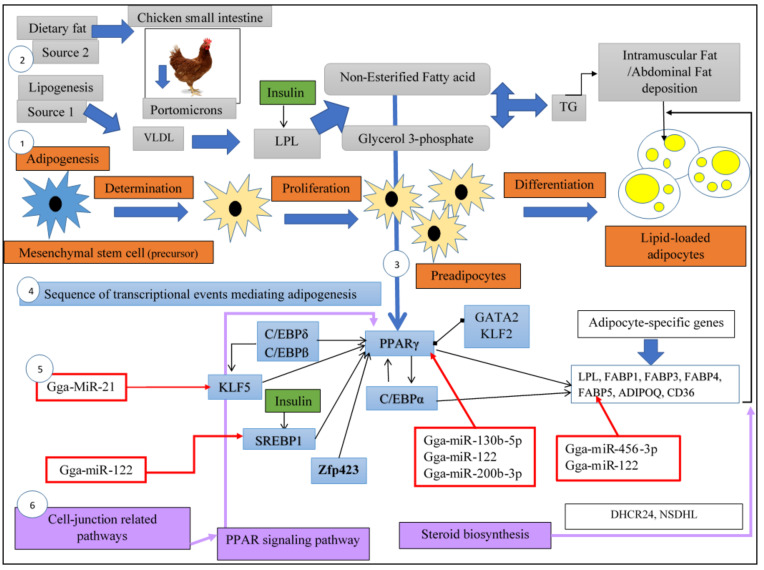
Schematic representation of adipogenesis and fat deposition and their regulation at the transcriptional and post-transcriptional level in the chicken adipocyte. Several regulatory factors, including hormones (green boxes), transcriptional factors (blue boxes), miRNAs (red boxes), and their target genes, controlling adipogenesis and fat deposition in chicken. 1—Adipogenesis (orange boxes) is the process of differentiation in which mesenchymal cells turn into mature adipocytes. 2—The fatty acids released from VLDL can penetrate adipocytes, which inside adipocytes resynthesized into TG and deposited. Accumulated fat in chicken adipose tissue consists of TG either from plasma VLDL (source 1) or from dietary fats (portomicrons) (source 2). 3—The presence of exogenous fatty acids (from VLDL or diet) is vital for regulating the expression of peroxisome proliferator-activated receptor-γ (PPARγ). 4—The blue boxes revealed the sequence of transcriptional factors controlling adipogenesis; the early transcriptional regulators, including CCAAT/Enhancer binding-protein (C/EBPβ and C/EBPδ) express and stimulate the key transcription factors, including PPARγ and C/EBPα which at terminal differentiation stage, regulate the transcriptional regulation of a variety of adipocyte-associated genes, such as lipoprotein lipase (LPL), fatty-acid-binding proteins (FABP), cluster of differentiation 36 (CD36), and adiponectin, C1Q and collagen domain-containing (ADIPOQ). PPARγ and C/EBPα are the master regulators of adipogenesis that can crossregulate each other. Sterol response element-binding protein (SREBP1) is regulated by insulin and can regulate adipogenesis by inspiration the expression of PPARγ through adipogenesis. Kruppel-like transcription factor (KLF5) is activated by C/EBPβ/δ through the early stages of adipogenesis and then promotes the expression of PPARγ. KLF2 and GATA binding protein 2 (GATA2) are the anti-adipogenic transcription factors, which lead to adipogenesis repression through inhibition of the expression of PPARγ. Zinc finger protein 423 (Zfp423) regulates preadipocyte cell determination through regulation of PPARγ expression. 5—The red boxes indicated various significantly expressed miRNAs with regulatory effects on adipogenesis. 6—Fat deposition in chicken is mediated and regulated by different signaling pathways (purple boxes), including the PPAR signaling pathway and the cell junction pathways (focal adhesions, tight junction, ECM-receptor interaction, regulation of actin cytoskeleton). The cell junction-related pathways may mediate the PPAR signaling pathway after activating PPARγ. Then PPARγ upregulates the expression of lipogenic-related genes, such as FABP3, LPL. The steroid biosynthesis pathway through regulating the expression of genes, such as 24-dehydrocholesterol reductase (DHCR24) and NAD(P) dependent steroid dehydrogenase-like (NSDHL), might play a dominant role in fat deposition in chicken (). Open arrows and diamond arrows represented the stimulating and inhibition effects, respectively.

The transportation of TG through VLDL to adipose and muscle tissues in chickens occurs similarly to mammals, in which the triglyceride-rich VLDL are catalyzed by insulin-activated LPL on endothelial cells lining the adipose tissue into VLDL remnants [31]. Afterward, the separated fatty acids from VLDL can penetrate adipocytes, where they are resynthesized again to TG and deposited [20] (Figure 2). It should be emphasized that the glycerol obtained from hydrolysis of TG in VLDL cannot be re-used for esterifying fatty acids, so the adipose tissue acquires the glycerol 3-phosphate for TG resynthesized from pyruvate or glucose through gluconeogenesis and glycolysis, respectively [31]. Moreover, in egg-laying hens, the VLDL catabolism is very limited in adipose and muscle tissue, so a greater quantity of lipids is transported to the oocytes, which supply lipids for the production of the egg yolk [20,33]. Fat deposition in chicken depends on the TG availability in plasma, which is transported to adipocytes as a component of lipoproteins; therefore, we can say that chicken fattening is related to the three aspects of lipid metabolism, including lipid synthesis, lipid transport, and lipid utilization [34]. Deposited fat in chicken mainly consists of abdominal fat, subcutaneous fat, and intramuscular fat (IMF). In which the IMF content in chicken relies on the number of adipocytes and the lipid deposition capacity [6].

### 2.2. Adipogenesis in Chicken

Adipose tissue development is a process comprises of adipocytes formation (adipogenesis) and the cellular accumulation of TG inside lipid droplets. On the other hand, adipose tissue development is a result of hyperplasia (proliferation of new adipocytes) and hypertrophy (increased accumulation of lipid in adipocytes) [31]. Besides, a significant amount of accumulated fat in chicken adipose tissue is the incorporated TG either from plasma VLDL or from dietary fats (portomicrons) [35,36,37] (Figure 2). Studies have revealed that the expression level of three transcription factors, including PPARγ, SREBP1, and C/EBPα, was dramatically promoted through preadipocyte differentiation, indicating that they have an important role in chicken adipogenesis by affecting the expression levels of target adipogenic genes [31,38,39]. Overall, adipogenesis in chicken comprises three different stages, including adipocyte proliferation, adipocyte differentiation, and lipid accumulation inside mature adipocytes. In which, several transcription factors, adipogenic-related genes, and miRNAs can regulate these three different stages through adipogenesis [40] (Figure 2). For instance, both transcription factors, including PPARγ, which participates in induction and stimulation of fat-specific genes and fatty acid synthesis, and PPARβ/δ, which participates in stimulation of fatty acid oxidation, are necessary to provide maximum lipid accumulation and differentiation during adipogenesis [35].

## 3. Important Genes Involved in Chicken Fat Metabolism

There are several genes involving in different stages of fat metabolism in chicken, which their expression is regulated via different miRNAs [27], transcription factors [11], and hormones [30]. Some of these genes, including ACACA, FASN, SCD, ELOVL6, and ACLY, encoding major enzymes catalyzing important steps in different lipid-related processes in chicken. Another group of genes, including -ACSL3, ACSL5, ACSL6, major facilitator superfamily domain-containing 2A (MFDS2A), and FABP7, involving fatty acid activation and transport. Furthermore, genes participate in the TG synthesis, remodeling, and packaging to VLDL for exporting to peripheral organs, including GPAT1, AGPAT2, LPIN1, DGAT2, and MTTP [24]. In the following parts, we summarized the characteristics of some of the most important genes involved in fat metabolism and deposition in chicken.

### 3.1. FABP

Fatty acid-binding proteins (FABP) are an important gene family that controls IMF content, meat tenderness, and flavor in chicken, and considered as a known-marker to identify IMF acceleration [41]. Moreover, FABPs are associated with lipid metabolism, including lipolysis and lipogenesis, homeostasis in adipocytes, and have been indicated as a main cytoplasmic protein related to glucose and lipid metabolic function. One of the FABP gene family members is FABP5, which transports intracellular fatty acids into the nucleus to activate PPARγ [23]. Another FABP member that is mainly expressed in the cardiac muscle, as well as skeletal muscle, and participates in the fatty acid transportation from cell membrane to the intracellular sites of fatty acid utilization, is FABP3 [6]. Various studies have revealed that FABP, along with other candidate genes, including PPAR, LPL, SCD, ACSL, and KLFs, have a significant role in IMF deposition and TG metabolism regulation in chickens [23]. Besides, FABP4, as an adipocyte differentiation marker, can regulate PPARγ expression and has an important role in fatty acid transportation and metabolism. Furthermore, its overexpression leads to hypertrophy by mediating the sequestration of fatty acids for TG synthesis [31].

### 3.2. GPAM

Glycerol-3-phosphate acyltransferase (GPAM) has a crucial role in the regulation of cellular TG and phospholipid levels. Moreover, GPAM along with FASN are the two main genes with central roles in de novo lipogenesis [42].

### 3.3. ELOVL

The ELOVL fatty acid elongase (ELOVL) gene family consists of seven isoforms (ELOVL1-7), which are the determinant of overall fatty acid elongation with variation in substrate specificity, tissue distribution, and regulation and are the vital regulators of cellular lipid composition [5]. Some members of the ELOVL family, such as ELOVL6, participates in the de novo lipogenesis in the chicken liver [42], and encodes for the ELOVL6 enzyme, which regulates the last step of the endogenous saturated fatty acid synthesis. Besides, in response to alterations in dietary and hormonal status in chicken, ELOVL6 regulation plays a vital role in controlling the hepatic lipid composition [5]. A newly discovered ELOVL protein family member is ELOVL7, which stimulating lipid accumulation in differentiated adipocytes. Moreover, the expression of ELOVL7 and SCD is higher in the breast muscle of chicken with the high TG content, which revealed that escalated synthesis of fatty acids may elevate the synthesis and deposition of TG [23]. Another member of this family, such as ELOVL5, is responsible for the hepatic synthesis of long-chain polyunsaturated fatty acids (LCPUFA) [13,43]. Furthermore, ELOVL5 is enriched in two important metabolic pathways, including biosynthesis of unsaturated fatty acids and fatty acid metabolism [44]. This gene plays a key role in the biosynthesis of omega-3 polyunsaturated fatty acids, including eicosapentaenoic acid (EPA) and docosahexaenoic acid (DHA), as well as omega-6 polyunsaturated fatty acids [15]. It is worth noting that the role of ELOVL5 in chicken is different from other poultry species, such as duck and turkey, which can elongate docosapentaenoic acid (DPA) to produce tetracosapentaenoic acid (24:5n-3) in chicken. Therefore, a chicken could be a dietary source of EPA and DHA in addition to fish. Besides, bioinformatics indicated that the ELOVL5 gene can be the putative target of miRNAs, including miR-19a-3p, miR-19b-3p, miR-30a-5p, miR-30b-5p, miR-30e-5p, and miR-218-5p, which can regulate its expression in chicken hepatocytes [44].

### 3.4. LPL

Lipoprotein lipase (LPL), which is expressed in several tissues, such as adipose tissue, provides instruction for coding a glycoprotein enzyme named lipoprotein lipase. Subsequently, the LPL enzyme participates in the hydrolysis of TG either from circulating chylomicrons or VLDL [6]. Moreover, it is indicated that LPL gene expression is significantly higher in fast-growing chickens compared to slow-growing chickens [42].

### 3.5. ACLY

ACLY encodes for the ATP citrate lyase, the primary enzyme connecting carbohydrate catabolism to lipogenesis by synthesizing cytosolic acetyl-CoA from citrate for both fatty acid and cholesterol biosynthesis [45]. In chicken, ACLY is among the numerous genes encoding crucial key enzymes in de novo fatty acid synthesis and is a direct target of SREBP1 as a major transcription factor of de novo lipogenesis regulation [24].

### 3.6. ACSL

Long-chain acyl-CoA synthetase (ACSL) is a candidate gene for the regulation of skeletal muscle fatty acid composition [15] and catalyzes the conversion of long-chain fatty acids into acyl-CoA [46]. ACSL1 as a dominating family member, is involved in multiple lipid metabolism pathways, including biosynthesis of unsaturated fatty acids, PPAR signaling, and fatty acid metabolism pathways [14]. ACSL5 is another family member that has a significant role in partitioning fatty acids towards TG, and its suppression could lead to lower fatty acid-induced lipid droplets formation [18]. Overall, ACSL3, ACSL5, and ACSL6 are involved in fatty acid activation, encoding key enzymes in fatty acid and TG synthesis pathways [24].

### 3.7. ACAA

It is revealed that acetyl-Coenzyme A acyltransferase (ACAA1 and ACAA2) are the genes that regulate IMF deposition in chicken breast muscle and take part in the fatty acid β-oxidation, as well as fatty acid degradation [15].

### 3.8. SCD

Stearoyl-CoA desaturase (SCD) gene, which is located on the GGA6 chromosome in chicken, encodes a key rate-limiting enzyme of de novo fatty acid synthesis with the ability to transform palmitic acid (C16:0) and stearic acid (C18:0) to palmitoleic (C16:1 n-7) and oleic acid (C18:1n-9), respectively [23,24]. Moreover, SCD influences several processes, such as lipid synthesis, lipid oxidation, and insulin sensitivity in liver, muscle, and adipose tissue in the mice. Besides, it may play a potential role in controlling body weight and energy homeostasis in chickens [14]. The deep RNA-sequencing of chicken with divergent unsaturated fatty acid contents in the breast muscle indicated that SCD, which is located through the genomic region of a quantitative trait locus for intramuscular and abdominal fat, can be regarded as a positional and functional candidate gene for the regulation of unsaturated fatty acid metabolism in chickens [33].

### 3.9. APOV1

Apovitellenin 1, also known as APOVLDLII is a kind of primary apoprotein of VLDL, synthesized in the liver and significantly stimulated by estrogen during sexual maturity [23]. It is revealed that APOV1 was downregulated in the liver of chicken with high unsaturated fatty acid content in breast muscle, which can lead to a considerable reduction in the level of cholesterol. While, in the liver of peak laying hens, the expression of this gene was more significant, boosting lipid metabolism, TG, cholesterol, and VLDL biosynthesis in the liver. At the same time, RNA sequencing of chicken liver with divergent unsaturated fatty acid content showed that APOV1 along with SCD could be potential candidate genes involved in fatty acid metabolism in the chicken liver [33].

### 3.10. ACACA

Acetyl-CoA carboxylase (ACACA) and FASN are the two important genes, encoding key enzymes in de novo fatty acid synthesis [24,47]. Acetyl-CoA carboxylase (ACC) is the enzyme encoded by ACACA, which catalyzes the conversion of acetyl-CoA to malonyl-CoA, the substrate of the de novo lipogenesis, and regulates the oxidation and synthesis of fatty acid. [26].

### 3.11. FAS or FASN

Fatty acid synthase (FASN) is another gene involves in de novo fatty acid synthesis in lipogenesis, which produces long-chain fatty acids in the presence of malonyl-CoA [24]. Interestingly, it is revealed that the expression of FASN increased by feed intake expansion in young chick which leads to increases in fatty acid synthesis in the post-hatch chicks [47].

### 3.12. FADS

Fatty acid desaturase 2 (FADS2) gene encodes for the enzyme, which is rate-limiting in the synthesis of long-chain polyunsaturated fatty acids [14]. It is indicated that FADS2 participates in both the PPAR signaling pathway and the biosynthesis of the unsaturated fatty acids pathway, which is targeted by the downregulated miR-30c-1-3p. Conversely, FADS1, which is enriched in the biosynthesis of unsaturated fatty acids, is targeted by several miRNAs, including miR-365-3p, miR-218-5p, miR-181a-5p, miR-181b-5p, miR-29a-3p, and miR-23b-3p, in the liver of laying hen chickens [18]. The integrated analysis of differential gene expression between broiler chicken with extremely high and low PUFA percentages of thigh muscle tissue revealed that FADS2 is one of the most significant candidate genes for PUFA percentage in chicken muscle [28].

## 4. Main Transcription Factors Involved in Chicken Fat Metabolism

In various species, particular transcription factors have significant roles in controlling adipogenesis, lipogenesis, and lipolysis. For instance, transcription factors, such as SREBP1 and C/EBPβ, regulate the early stages of adipogenesis in chicken. The main transcription factors related to fat metabolism and deposition in chicken consist of SREBP1, C/EBPβ, C/EBPα, PPARγ, and PPARα [48], which are briefly introduced in the following parts.

### 4.1. SREBPs (SREBFs)

Sterol regulatory element-binding proteins (SREBPs) are the hepatic transcription factors that can activate genes required for fatty acid synthesis and indirectly regulate different processes, such as cholesterol biosynthesis, fatty acid uptake, and fatty acid biosynthesis [3]. One of the most important members of the SREBPs family is SREBP1, which its overexpression can elevate fatty acid secretion and fatty liver. In chicken, SREBP1 regulates lipid metabolism at the early developmental stages. It is well demonstrated that SREBP1 regulates lipogenesis through targeting PPARγ and C/EBPα transcription factors [48]. Besides, SREBP1 plays significant roles in regulating the expression of lipogenic genes involved in fatty acid and cholesterol synthesis, such as FASN, in skeletal muscle, liver, and adipose tissue [49]; as well as genes encoding the enzymes involved in FA and TG syntheses (key lipogenic enzyme), such as ACLY, ACACA, FASN, ELOVL6, and SCD [31] (Figure 1). Besides, the dietary PUFAs in the chicken with a high-fat diet inhibit SREBP1 activity through different mechanisms. Therefore, dietary PUFAs can decrease de novo fatty acid synthesis and TG secretion in the chicken liver through downregulation of SREBP1 and lipogenic genes [24]. SREBP1, as one of the adipocyte-related transcription factors, is significantly expressed in adipose tissue and has an important role in adipocyte development by promoting the expression of PPARγ, generation of an endogenous PPARγ ligand, and the expression of several genes critical for lipid biosynthesis [17] (Figure 2).

### 4.2. C/EBPβ

The expression of CCAAT Enhancer Binding Protein β (C/EBPβ) in the breast muscle and abdominal fat of broiler chickens was significantly higher at early developmental stages (4 and 8 weeks) compared to the late developmental stage (14 and 20 weeks) and positively correlated with the IMF content of chicken breast and thigh muscle. It is deduced that C/EBPβ plays an important role in lipid accumulation in adipocyte differentiation rather than in fat synthesis. Moreover, its expression in mature fat cells is directly regulated by insulin and other transcription factors, such as SREBP1 [48]. C/EBPβ as an early transcriptional regulator of adipogenesis, inducing the expression of PPARγ and C/EBPα as key transcription factors organizing the regulation of a variety of adipocyte-associated genes [31] (Figure 2).

### 4.3. C/EBPα

CCAAT/Enhancer Binding Protein α (C/EBPα) along with PPARγ and SREBP1 are the three-adipogenic transcription factors and play a vital role in chicken adipogenesis (Figure 2). C/EBPα has a vital role in the transcriptional activation of adipocyte differentiation by stimulating lipid-specific genes that are required for the synthesis, uptake, and storage of long-chain fatty acids [31] (Figure 2). Moreover, this transcription factor is mainly expressed in adipose tissue and the liver [39]. (Figure 2).

### 4.4. PPARγ

Peroxisome proliferator-activated receptors (PPARs) are ligand-activated transcription factors, which have three isoforms (α, β or δ, γ) and belong to the nuclear hormone receptor superfamily [50]. Among PPARs, PPARγ involves in adipose development and function, and it is the key regulator in chicken adipogenesis [31] (Figure 2). PPARγ gene expression begins in the early stage of preadipocyte differentiation [51], which has a role in the adipocyte differentiation program during the adipogenesis process [26]. Previous studies have indicated that PPARγ predominantly expressed in adipocytes and in combination with other transcription factors, such as C/EBPα, participating in fat cell development pathways and stimulating the expression of genes related to lipid metabolism and adipocyte differentiation in chickens [39] (Figure 2). In fact, C/EBPβ at the later stages of cell differentiation promotes the expression of C/EBPα and PPARγ, which are factors that play a vital role in the differentiation of adipocytes, lipid synthesis, and other related processes to fat metabolism [31]. In chicken, the expression of these two transcription factors is significantly higher in abdominal fat compared to breast and thigh muscles, which revealed that the abdominal fat tissue has significantly higher fat deposition compared with breast and thigh muscles. Furthermore, the expression of C/EBPα is positively correlated with the expression of PPARγ in chicken breast and thigh muscle, revealing that these two transcription factors promoted fat deposition in chickens [48]. Moreover, PPARγ and FABP4 are the popular adipogenic markers, which significantly upregulated during adipocyte differentiation [52]. The study of the molecular regulatory mechanism of IMF metabolism in the chicken breast and thigh muscles indicated the downregulation of PPARγ, LPL, and FABP4 genes and upregulation of retinoid X receptor α (RXRA) and C/EBPβ in breast muscle with the lower IMF content, compared with thigh muscle. On the other hand, transcriptome analysis of abdominal fat in chickens with divergent growth rate and abdominal fatness demonstrated that overexpression of many transcription factors, such as PPARγ, and its target genes lead to increased synthesis and storage of fatty acids in abdominal fat of high growth chicken. In contrast, PPARδ by inhibiting the expression of genes, such as SCD and FASN, can prevent lipid synthesis while promoting lipolysis and fat catabolism in the low growth chicken [36]. Other investigations indicated that suppression of PPARγ could inhibit the chicken preadipocyte differentiation and promote proliferation which shows the important role of this transcription factor in chicken adipogenesis [31]. In addition, fatty acid as an important inducer of chicken adipocyte differentiation, act as a PPAR ligands and increases PPARγ gene expression [31] (Figure 2). Therefore, it should be noted that exogenous fatty acids are important in chicken adipogenesis and fat accumulation [35].

### 4.5. PPARα

Liver and brown adipose tissue are the main places for PPARα expression [48]. PPARα is a transcription factor with the ability to enhance fatty acid β-oxidative and lipid oxidation [35]. It is revealed that PPARα is negatively correlated with IMF content in thigh muscle and its expression level in thigh muscle is higher than abdominal fat in chicken, which is consistent with the fact that fat accumulation in the thigh is lower than abdominal fat [48].

### 4.6. PPARβ/δ

This transcription factor is another member of PPARs, which in comparison with PPARγ, stimulates fatty acid oxidation and leads to increase energy production in the form of adenosine triphosphate (ATP). Various studies revealed that both PPARβ/δ and PPARγ are necessary for lipid accumulation and differentiation during adipogenesis. Moreover, both of these transcription factors need fatty acids for activation, which then are stimulated by SREBP1 in adipocytes during adipogenesis (Figure 2). It should be noted that PPARβ/δ has no role in chicken adipocyte differentiation, but it can promote the transformation of chicken adipocytes to mature adipocytes [35].

### 4.7. ZFP 423

The zinc finger protein 423 (ZFP 423) is considered a preadipocyte cell determination regulator. This transcription factor is abundantly expressed in preadipocytes compared with non-preadipocyte fibroblast [31]. Moreover, ZFP 423 takes part in the initial stages of adipogenesis, which is the transition of stem cells into a preadipocyte [22] (Figure 2).

### 4.8. KLFs

Kruppel-like transcription factors (KLFs) are another family considering as the key regulators of adipogenesis [53]. Among KLFs family members, KLF5 is activated by C/EBPβ and C/EBPα, and, together with these two transcription factors, participating in the induction of PPARγ expression. Therefore, it can affect adipogenesis by regulating the adipocytes proliferation [31,53] (Figure 2). Other KLF family members, such as KLF6 and KLF15, capable of promoting adipogenesis. In contrast, some of the KLF members, such as KLF2, are anti-adipogenic, which binding suppresses transcription from the PPARγ promoter (Figure 2). KLF7 is another member that promotes chicken preadipocyte proliferation, but represses its differentiation [31]. Overall, investigations have demonstrated that KLFs 2, 3, and 7 inhibit chicken preadipocyte differentiation and that KLF 7 along with KLF 5 promote chicken preadipocyte proliferation. Thus, KLF5 and KLF7 are apparently involved in regulating preadipocyte proliferation and differentiation [53].

### 4.9. GATA2

GATA-binding factor2 (GATA2) is a preadipocyte-specific transcription factor that its overexpression inhibits preadipocyte differentiation in chicken, perhaps through inhibition of the expression of C/EBPα and PPARγ transcription factors [31] (Figure 2).

There are several other transcription factors whose differential expression has a significant impact on the growth rate and abdominal fat content phenotypes. Accordingly, overexpression of regulators, including thyroid hormone-responsive Spot 14 protein, α (THRSPA), E2F transcription factor 4 (E2F4), FOXO1, KLF9, KLF13, PPARγ, SREBF1, SREBF2, and C/EBPα by controlling the expression of lipogenic genes, including FADS2, FASN, SCD, and DGAT2, led to increased abdominal fatness and body weight phenotypes in chicken. While, the upregulated transcription regulators that led to diminishing body weight and abdominal fatness were PPARδ, C/EBPβ, KLF5, and signal transducer and activator of transcription 5B (STAT5B) [36].

## 5. Chicken miRNAs

MiRNAs as an abundant short (16–26 nt) single-stranded noncoding RNAs in the animal are the gene expression regulators, which regulate the expression of target genes, post-transcriptionally by binding to specific sites in the three prime untranslated regions (3′-UTR) of their target mRNAs [52], through one of the two post-transcriptional mechanisms, including translational repression or mRNA degradation [54]. Since miRNAs have differential expression levels in various tissues, growth and physiological stages, as well as chicken breeds, to date miRNAs in chicken have been investigated in a variety of chicken tissues (breast muscle, liver, adipose tissue, etc.), different ages, and physiological stages, as well as different chicken breeds with a combination of different methods [15,55]. So far, many miRNAs with different biological effects have been reported in chicken which most of which are similar to miRNAs that are phylogenetically conserved in vertebrates except for miR-757, which is the chicken-specific miRNA [34,55]. Furthermore, the miRNA expression pattern in chicken is in accordance with the developmental stages of the chicken. For instance, it is well documented that miRNAs cannot be completely detected at the early developmental stages in the chicken embryo, but at different stages of later development, a variety of miRNAs emerge with diverse expression patterns. Moreover, miRNAs are not randomly distributed throughout the genome. In fact, the majority of chicken miRNAs (66%) are intergenically encoded, while about 33% of miRNAs genes hosted within the introns [55]. Besides, some miRNAs in the chicken are extremely tissue-specific, such as miR-122, miR-132, and miR-338, which are only expressed in the liver, cerebrum, and cerebellum of chicken, respectively [56].

### 5.1. MiRNAs in Regulation of Chicken Fat Metabolism

Genetic background is responsible for almost 25–70% of fat distribution changes through the human and animal body [27]. Recent studies have indicated that miRNAs play a significant role in adipose tissue growth and lipid metabolism in chickens. In fact, the potential targeting of genes by miRNAs will add an additional layer to the complexity of lipid metabolic regulation in chicken at the post-transcriptional level [47,57]. In this section, we summarized recent investigations regarding the significant contribution of specific miRNAs in the regulation of lipid metabolism, including lipogenesis, adipogenesis, and intramuscular/abdominal fat deposition in chickens.

#### 5.1.1. MiRNA-Mediated Regulation of Lipogenesis (Hepatic Lipid Metabolism)

To characterize the role of miRNAs in chicken liver, which are involved in lipogenesis, it is crucial to identify miRNAs that significantly express in chicken liver. Subsequently, the focus can then be placed on determining the function of specific miRNAs regulating lipogenesis [47] (Figure 1). Since hepatic lipid metabolism relates closely to adipose fat deposition [34], affects economic traits of chickens and consumer’s health [58], knowing the molecular regulatory mechanism of lipogenesis and fatty acid synthesis in chicken can help us to shed light on the divergent fat content in various chicken groups [57]. In the following sections, we mentioned some of the most important miRNAs, which regulate lipogenesis in chickens.

##### Gga-miR-33

The miR-33 family is the well-known miRNA that participates in the regulation of lipid metabolism. This family contains two isomers, including miR-33a and miR-33b, which are different by two nucleotides in their natural forms. MiR-33a and miR-33b are encoded by the intron region of the SREBP2 and SREBP1, respectively [59]. MiR-33a is significantly conserved throughout evolution, while miR-33b is mostly expressed in large mammals, but not in a chicken. It is revealed that intron 16 of the SREBF2 gene encodes a miR-33, which affects the activation of many genes involved in the synthesis and uptake of cholesterol, fatty acids, TG, and phospholipids [3]. Chicken miR-33 may have a dominant role in lipid metabolism and energy hemostasis by downregulating the expression of fat mass and obesity-associated gene (FTO) in chicken hepatocytes, which in human FTO is strongly associated with obesity. Moreover, the expression of miR-33 was escalated from day 0 to day 49 of age in chicken liver, while the expression of the FTO gene was decreased during this period of chicken growth. So, it can be concluded that miR-33 plays a significant role in lipid metabolism and energy homeostasis by negatively regulating the expression of the FTO gene in the chicken liver [60]. Other studies have shown that miR-33 involves in lipid metabolism and controls fatty acid oxidation in the chicken liver by negatively regulating the carnitine o-octanoyl transferase (CROT) and hydroxy acyl-CoA dehydrogenase subunit β (HADHB) genes in the liver, which encodes enzymes vital for lipid oxidation [3]. The characterization of miRNA expression in the embryonic chick liver at the liver developmental stage (embryonic day 15 and 20) indicated that a differentially expressed novel miRNA, nc-miR-33, is one of the liver-associated miRNAs that can potentially regulate FASN gene expression as one of the key genes in lipogenesis [47]. Furthermore, miR-33 reduces fatty acid degradation by targeting multiple genes involved in fatty acid β-oxidation [13]. MiR-33 plays an important role in liver metabolism by regulating lipid homeostasis and cholesterol metabolism by targeting genes involved in cholesterol transport and reduces fatty acid degradation by targeting genes related to fatty acid β oxidation in the liver [61]. Therefore, miR-33a and miR-33b, with their encoding gene SREBP, are important regulators of genes involved in lipogenesis [18].

##### Gga-miR-122

The miRNA expression analysis in the liver of embryonic chicks shows that 30% of total reads belonged to miR-122 that is the most highly expressed miRNA in the liver [56], followed by miR-126, miR-17-5p, and miR-19b. Moreover, miR-122 is highly conserved in vertebrates and involved in regulating cholesterol synthesis and lipid metabolism [47]. It is revealed that miR-122 has a significant role in the growth and development of the liver, lipogenesis, and also liver diseases [62]. Although several reports showed that miR-122 is the most abundant miRNA in the chicken liver [61], some other studies considered miR-148a as the high abundance of miRNA in chicken hepatocytes [13], which demonstrates the importance of breed, developmental stages, sex, and age of chickens on the abundance of specific miRNAs [15]. Investigations have revealed that Vanin 1 (VNN1) gene, which encodes a glycosylphosphatidylinositol (GPI) and plays an important role in hepatic lipid metabolism, contains a binding site in 3′-UTR for binding miR-122. Furthermore, VNN1 expression is downregulated by miR-122 in chicken hepatocytes; therefore, miR-122 is involved in lipid metabolism and energy homeostasis in the chicken liver by negatively regulating the expression of the VNN1 gene [61,62]. Other target genes of miR-122 in chicken liver, which are known to play roles in lipid metabolism, are transforming growth factor-β 3 (TGFB3), FABP5 (Figure 2), and archain 1(ARCN1) [62]. The expression pattern of miR-122 in different chicken tissues suggested that chicken miR-122 is the abundantly expressed miRNAs in the liver, but it also can be detected in some extra-hepatic tissues, such as chicken lung, kidney, and adipose tissue [61]. Furthermore, the expression of miR-122 escalates progressively from hatch to 7 weeks of age in chicken liver, implying it might play an important role in the growth or functional maturation of the liver in chickens [62]. The finding revealed that miR-122 has a significant role in liver metabolism, especially in lipid metabolism, by directly or indirectly targeting genes, such as pyruvate kinase M2 (PKM2), TGFB3, FABP5, ARCN1, FASN, SCD, ACACA, prolyl 4-hydroxylase, α polypeptide I (P4HA1), SREBP1, and SREBP2, which are known to have a role in lipid metabolism [61]. Collectively, it can be concluded that miR-122 is a liver-specific miRNA that can regulate fatty acid and cholesterol synthesis, as well as hepatic fatty acid oxidation [18] (Figure 1 and Figure 2).

##### Gga-miR-5

Another miRNA identified in miRNA profiling of the embryonic chicks is miR-5, which can target 3′-UTR of transcription factor CAMP responsive element binding protein 1 (C/REB1) to regulate CREB-mediated hepatocyte growth, as well as PPAR-binding protein (PBP/PPARBP), which is the coactivator in PPARα signaling pathway. Therefore, this miRNA affects hepatocyte proliferation [47].

##### Gga-miR-22-3p

MiRNA-22-3p is another abundant miRNA expressed in the liver of egg-laying hens, which targets several genes involved in chicken hepatic fatty acid metabolism, including ACSL5, ELOVL6, and PLIN2. Moreover, miR-22-3p may be involved in lipid accumulation in the chicken liver by binding to target genes involved in the hepatic fatty acid metabolism. The miRNA-mRNA interaction revealed that miR-22-3p involves in various pathways, such as unsaturated fatty acid biosynthesis and PPAR signaling pathways, by targeting ELOVL6 and ACSL5 genes [18]. Another study indicated that the expression level of ELOVL6 in the liver of pre- and peak laying hens was negatively regulated by miR-22-3p, which can be considered as an important regulator of the hepatic lipid metabolism during the egg-laying stage in hens. Subsequently, the expression level of miR-22-3p significantly decreased in the liver of peak laying hen while ELOVL6 gene was significantly upregulated in the liver of peak laying hens compared with prelaying hens, which can confirm the negative regulatory effect of miR-22-3p on ELOVL6 expression level in the chicken liver in two stages [5,18] (Figure 1).

##### Gga-miR-146b-5p

Mir-146b-5p is another miRNA related to hepatic lipid metabolism, which is located in an intergenic region of chicken chromosome 6, and it is highly conserved in most vertebrate species, including mammals [18]. In chicken, miR-146b-5p was upregulated in the breast muscle of rapid growth broiler compared to slow-growth chickens. Therefore, it can be considered as breast muscle-specific miRNA, which can lead to rapid growth and higher feed efficiency in modern broiler chickens [63]. The expression level of miR-146b-5p in egg-laying hens at 30 weeks showed an 8.50-fold change compared to 20 weeks [18]. Since previous studies have revealed that this miRNA also can promote adipogenesis in chickens [15], it can be concluded that miR-146b-5p can directly affect lipid metabolism in the chicken liver [18].

##### Gga-miR-24-3b

Studies revealed that miR-24-3p is involved in both chicken muscle development and fat deposition [15]. For instance, in the liver of egg-laying hens, miR-24-3b was found to be downregulated about 7.39-fold in 30-week chicken to maintain hepatic lipid homeostasis. Therefore, the significant increase in lipid accumulation in the liver of 30-week old chicken can be explained by the downregulation of mir-24-3b in the liver of this chicken [18].

##### Gga-miR-34a

MiR-34a-5p, a member of the miR-34a family, is responsible for increasing the intracellular levels of TG, as well as total cholesterol in hepatocytes by inhibiting the translation of its target gene, ACSL1, in chicken livers. It is indicated that miR-34a-5p has a significantly higher expression level in the liver of peak laying hens compared with prelaying hens and closely related to lipid metabolism [46]. Besides, the differential expression analysis of miRNAs in Gushi chicken breast muscle at different development stages showed that miR-34a-3p is a stage-specific miRNA that was abundantly expressed at 30 weeks of chicken age [15].

##### Gga-miR-218-5p

The study of Zhang et al. revealed that the ELOVL5 is highly expressed in the liver of peak laying hens compared to prelaying hens. At a subsequent time, the expression level of miR-218-5p was negatively associated with the mRNA level of ELOVL5 in the liver of hens. Since ELOVL5 is a putative target of miR-218-5p (Figure 1), overexpression and knockdown of miR-218-5p in hepatocytes significantly lead to up- and downregulation of ELOVL5 expression in the liver [44]. The miRNA-mRNA network analysis showed that FADS1, which has a significant role in the long-chain polyunsaturated fatty acids biosynthesis process, was a target gene of miR-218-5p [18] (Figure 1). Other integrated analysis studies based on the chicken breast muscle transcriptome data showed that a large number of differentially expressed genes related to lipid metabolism, such as ELOVL2,5, and 6, were significantly targeted with upregulated miR-218-5p in the Gushi chicken breast muscle [7] (Figure 1).

##### Gga-miR-101-2-5p

MiR-101-2-5p and Apolipoprotein (ApoB) are significantly down- and upregulated, respectively, in the liver of peak laying hen compared with prelaying hens [44]. Therefore, it can be concluded that miR-101-2-5p might be involved in lipid metabolism by negatively regulating the expression of ApoB, which plays a vital role in the assembly and secretion of TG-rich lipoprotein in the liver of egg-laying chickens [64].

Other important-significantly expressed miRNAs associated with lipogenesis in the chicken liver can be seen in (Table 1).

#### 5.1.2. MiRNAs Involved in Adipogenesis and Fat Deposition

A number of studies have indicated that miRNAs take part in the biological processes involved in adipose tissue development, such as adipocyte proliferation and differentiation and lipid metabolism. Moreover, miRNAs have been novel targets for inspecting the molecular mechanism related to abdominal and intramuscular adipose tissue development in animals [65]. In fact, IMF is an important determinant of meat quality in chicken by affecting tenderness, juiciness, and flavor of the chicken. Therefore, investigating the molecular regulation mechanism of IMF deposition in chicken muscles, particularly breast muscle, is an important consideration in the area of chicken meat quality and production [52]. Genetic, nutrition, and environment are the influential factors that regulate the chicken IMF deposition process, which in terms of genetic factors, the regulatory mechanism of miRNA in chicken intramuscular adipogenesis is still poorly investigated [57]. On the other hand, genetic selection in meat-type chickens for an increased growth rate has been led to excessive fat accumulation, particularly in the abdominal cavity, which has unfavorable effects on meat quality properties [36]. Abdominal fat deposition in chicken is regulated by multi-genetic factors, including miRNAs, endocrine hormones, and environmental factors [2]. Thus, understanding the molecular regulatory mechanisms behind abdominal adipose tissue development provides useful insight into biomedical research, and it can be beneficial for the chicken breeding and production sector [1,36]. In meat-type chickens, divergent genetic selection for bodyweight has extensive effects on abdominal fatness [36]. Therefore, investigating the transcriptional and post-transcriptional regulatory mechanism underlying abdominal and intramuscular adipose tissue development in chickens is very important and helps to increase the productivity of the chicken industry, improve meat quality [27], and provide new strategies for dealing with the excessive accumulation of lipids in adipose tissue [66]. In this section, we summarized the effect of significant miRNAs handling the regulation of chicken abdominal and intramuscular adipogenesis in chicken.

##### MiRNA and Abdominal Fat Adipogenesis in Chicken

In the process of chicken abdominal adipose tissue development and fat deposition, there is a complicated regulatory network created by the interactions between miRNAs and their target gene, as well as different pathways modulating chicken abdominal adipose tissue development [27].

The miRNA expression profiling, as well as miRNA-mRNA interaction analysis of abdominal fat in female chicken with divergent high and low abdominal fat content, revealed that differentially expressed miRNA, including miR-142-3p, miR-19a-3p, miR-19b-3p, miR-30d, miR-26a, miR-17-5p, miR-103-3p, miR-27b-3p, and miR-92-3p, miR-122-5p and genes ACSL1, FADS2 and ATP-binding cassette, sub-family D, member 3 (ABCD3) are the most important factors regulating fat metabolism and accumulation of abdominal fat in chicken. Among the aforementioned miRNAs, miR-30d and miR-26a are downregulated miRNAs in chickens with higher abdominal fat content and play important roles in lipid metabolism in chickens [14]. It is revealed that miR-30d, along with miR-30a-5p, miR-146b-5p, miR-21, and miR-101-3p, were abundantly expressed in chicken abdominal adipose tissue and have a role in adipogenesis regulation [27]. Moreover, the miR-17-92 cluster that is comprised of various miRNA members is associated with lipid metabolism and adipogenesis [27,52]. For instance, miR-19b-3p, as a member of the miR-17-92 cluster, is highly expressed in the chicken with high abdominal fat content and significantly enhanced adipogenesis and leads to the increased accumulation of abdominal fat by downregulating the ACSL1 gene [14] (Figure 1). In another study, 51 significant differentially expressed miRNAs were recognized in Gushi chicken (Chinese domestic chicken) by sequencing four RNA libraries constructed from abdominal adipose tissue during chicken postnatal late development stages (6, 14, 22, and 30 weeks). The expression pattern of these miRNAs was different among different developmental stages and revealed the variation in the miRNA expression profiles in chickens during different stages of abdominal fat development. Furthermore, the results of miRNA sequencing demonstrated that the most abundantly expressed miRNAs in Gushi abdominal adipose tissue is let-7 family (specifically let-7a-5p, let-7b, let-7f-5p, let-7g-5p, let-7i, and let-7k-5p), which were expressed highly in an abdominal fat at all development stages [27]. The result of this study is consistent with the study of Wang et al., who showed that the let-7 miRNA family, specifically gga-let-7a and gga-let-7j, were the most abundantly expressed miRNAs between chickens with divergent abdominal fat content [65]. Let-7 miRNA family is one of the most conserved miRNAs family in animals ranging from invertebrates to vertebrates indicated to be upregulated during adipogenesis and involved in fat deposition and muscle development in chickens [15]. Other most abundant expressed miRNAs in chicken abdominal adipose tissue are miR-148a-3p [15,27,65], and miR-17 family, such as gga-miR-17-3p [27,65], which significantly differentially expressed between chicken lines with high and low abdominal fat content [65]. Other high-abundance and significant differentially expressed miRNAs in chicken abdominal fat are summarized in (Table 2). Subsequently, gene ontology analysis revealed that the significantly differentially expressed miRNAs in preadipocytes of two broiler line with divergent abdominal fat content were enriched in pathways predominantly related to preadipocyte development and lipid metabolism, including mesenchymal cell differentiation, chromatin regulator, regulation of programmed cell death, and the cell morphogenesis, as well as a cellular response to insulin stimulus, and insulin-like growth factor 1 (IGF-1) signaling pathway. It is worth noting that IGF-1, as one of the adipocyte differentiation stimulators, has been revealed to be related to abdominal fat content and growth in chickens [65]. Furthermore, the miRNAs expression assay in chicken abdominal fat revealed that miR-122 is the adipose-related miRNA that its expression in abdominal adipose tissue has an opposite trend to PPARγ. Since PPARγ has a binding site for miR-122 and is important to the growth and development of adipose tissue, it can be concluded, that miR-122 may play an important role in the growth and development of not only the liver, but also adipose tissue [65] (Figure 2). On the other hand, during adipose tissue development in chicken, miR-200b expression has a decreasing trend, which suggested that this miRNA plays an important role in early adipocyte developmental stages rather than late stages [65]. MiRNA expression profile of chicken abdominal fat preadipocytes indicated that the most frequently expressed miRNA in chicken preadipocyte was miR-222, which most probably is a negative regulator of adipocyte differentiation in chicken. Other frequently expressed miRNAs in chicken preadipocytes were let-7 family members, miR-30, miR-26a, miR-21, miR-103, and miR-181a [66]. Similarly, previous studies showed that miR-30 [27], and miR-21 [27,65] were the highly expressed miRNA after the let-7 family in abdominal adipose tissue of broiler chickens [65] and Gushi chickens [27], which may have a regulatory effect on adipogenesis. Among the aforementioned miRNAs, miR-21is plentifully expressed in preadipocytes and inhibits chicken preadipocyte proliferation by targeting and inhibiting the KLF5 transcription factor [61] (Figure 2). Interestingly, for the first time, Ma et al. have created the differentiation model of chicken (Gushi) abdominal fat cells in vitro to indicate key miRNAs that regulate chicken abdominal fat deposition [37]. In this regard, the differential expression analysis of miRNA between abdominal preadipocytes (Ab-Pre) and differentiated adipocytes (Ab-Ad) revealed that 10 miRNA, including gga-miR-101-3p, gga-miR-10a-5p, gga-miR-146b-5p, gga-miR-199-3p, gga-miR-1559-5p, gga-miR-214, gga-miR-30a-3p, gga-miR-106-3p, gga-miR-458a-3p, and gga-miR-148a-5p, were significantly expressed in both library but in different rate. For instance, miR-10a-5p was upregulated in Ab-Ad in comparison with Ab-Pre. Moreover, some of these significant differentially expressed miRNAs, such as miR-214 and miR-10a-5p, by targeting genes ACLY and ELOVL5, respectively, and participating in different signaling pathways related to lipid metabolism, such as PPAR signaling, biosynthesis of unsaturated fatty acids, insulin signaling pathways, etc., have a strong association with abdominal adipocyte differentiation in chickens [37].

##### MiRNA and Intramuscular Adipogenesis in Chicken

IMF content is the predominant factor affecting chicken meat quality. Therefore, there is a need to know about molecular and post-transcriptional regulation mechanisms of different miRNAs on chicken IMF deposition and intramuscular adipocyte differentiation [7]. IMF is the deposited fat in the muscle tissue, which is distributed in the epimysium, perimysium, and endomysium, as well as in the form of lipid droplets in the muscle cytoplasm. Besides, it is composed of an intracellular phospholipid, TG (main component), and cholesterol [18,57]. IMF deposition is a dynamic process that consists of series of steps, including adipocyte differentiation, as well as synthesis, transportation, and decomposition of lipids [6]. Previous studies have revealed that miRNAs are significantly associated with fat deposition, adipocyte differentiation, and meat quality in chickens [7]. As an example, the miRNAs expression profile of breast muscle in Gushi chicken indicated that miRNAs, such as gga-miR-103-3p and miR-138-3-3p, have key roles in IMF deposition [15]. It should be pointed out that the physiological characteristics of chicken will determine the pattern of miRNA expression and fat deposition in chicken breast muscle [7,15]. In fact, the different expression patterns of miRNAs in different developmental or physiological stages may affect IMF deposition in chicken breast muscle [15]. For instance, the IMF content of chicken breast muscle gradually decreases before sexual maturity, due to rapid growth and high energy consumption, while it progressively increases after chicken sexual maturity. Likewise, the expression level of miRNAs, such as gga-miR-10b, gga-miR-103, gga-miR-27b, gga-miR-155, and gga-miR-133b, which are known to involve in fat deposition, increased significantly in breast muscle after chicken sexual maturity [15]. On the other hand, the study of Zhang et al. revealed that there were significant differences in IMF content and lipid metabolism levels between juvenile (20 weeks old) and late-laying-period (55 weeks old) hens. The high-throughput miRNA sequencing of breast tissue of these chickens in different ages showed that a total of 104 miRNAs significantly differentially expressed among these two groups. In which, late-laying-period hens, with higher IMF content, had lower expression levels of miRNAs than juvenile hens. Moreover, among these differentially expressed miRNAs, gga-miR-140-5p by targeting retinoid X receptor γ (RXRG) promoting intramuscular adipocyte differentiation [7]. Various factors that have an impact on both muscle and adipose tissue development also can affect intramuscular fat deposition in muscle, specifically in the embryo and through early growth [52]. For instance, the rapid growth in the diameter of the breast muscle fibers until 22 weeks of age led to decreased IMF content, which is subsequently dramatically increased after the diminished muscle fiber growth rate [15]. Other important miRNAs that regulate intramuscular fat deposition and development are summarized in Table 3 and explained in the following paragraphs.

##### Gga-miR-223

**Figure 3:**
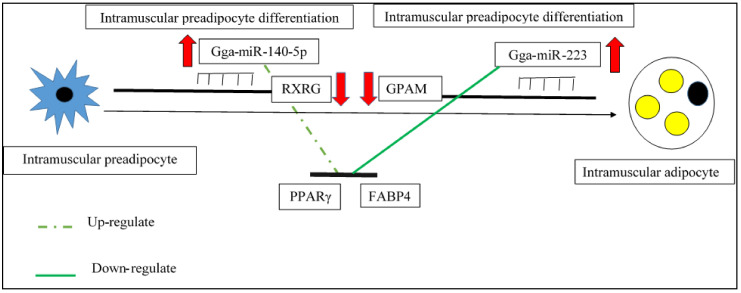
Differentially expressed miRNA through chicken intramuscular preadipocyte differentiation in the breast muscle between juvenile and late-laying hen. Overexpression of gga-miR-140-5p reduced the expression of retinoid X receptor γ (RXRG) while upregulated the expression of adipogenic markers, including the peroxisome proliferator-activated receptor γ (PPARγ) and fatty acid-binding protein 4 (FABP4); promoting intramuscular preadipocyte differentiation and IMF deposition in chickens [7]. Controversy, overexpression of gga-miR-223, as a negative regulator of chicken intramuscular preadipocyte differentiation, significantly reduced the expression of its target gene glycerol-3-phosphate acyltransferase (GPAM), as well as PPARγ and FAPB4, and lead to intramuscular preadipocyte differentiation inhibition and decrease the number of IMF droplets in the adipocyte [52].

MiR-223 is a highly conserved miRNA that regulates adipocyte differentiation by targeting genes associated with lipid metabolism [52]. The investigation to find miRNAs affecting the differences in meat quality between the two age stages of laying hens showed that miR-223 was significantly downregulated in breast muscle of late-laying hen with higher IMF fat. Therefore, it can be concluded that miR-223 may be associated with IMF adipogenesis [7]. Furthermore, bioinformatics analysis indicated that the mitochondrial glycerol-3-phosphate acyltransferase (GPAM) gene, which promotes the synthesis of TG in the fat metabolism pathway, is the putative target of miR-223. Subsequently, miR-223 negatively regulates the chicken intramuscular preadipocyte differentiation by targeting the GPAM gene (Figure 3), and its overexpression in the intramuscular preadipocytes leads to a downregulating the expression of GPAM. Thus, it can lead to lower TG synthesis, as well as the reduction in the numbers of lipid droplets in the adipocytes. While knockdown of miR-223 increases GPAM expression and the lipid droplet abundance in the adipocytes. For instance, miR-223 is significantly downregulated in the breast muscle tissues of Gushi hens at their later-laying period compared with the prelaying period, which shows differences in lipid metabolism in hens at the pre- (20 weeks old) and peak laying stages (30 weeks old) [52] (Figure 3).

##### Gga-miR-140-5p

Since there is numerous differentially expressed genes associated with meat quality and remarkable differences in the meat traits, including the IMF content of breast muscle between chickens with far divergent content, it can be concluded that various miRNA may consider as important candidate regulators of chicken meat quality [7,52,57]. The miRNA expression profiling of breast muscle in late-laying and juvenile hen for investigating the mechanism underlying differences in meat quality indicated that the majority of differentially expressed miRNA, including miR-222-3p, miR-222b-5p, miR-9-3P, mir-206, and miR-140-5p, were downregulated in late-laying hens. Therefore, it can lead to the conclusion that these miRNAs may play an important role in gene regulation related to lipid metabolism and deposition. Among these downregulated miRNA, gga-miR-140-5p was greatly expressed in abdominal fat, and its level raised through intramuscular preadipocyte differentiation. In fact, it is revealed that this miRNA is a novel promoter of chicken intramuscular adipogenesis, negatively regulating the expression of the RXRG gene (Figure 3). Besides, overexpression of gga-miR-140-5p increased the expression of the adipocyte markers, including PPARγ and FABP4, but decreased the expression of RXRG and forkhead box O1 (FOXO1) [7] (Figure 3). However, a study by Sun et al. [57] considered gga-miR-18b-3p instead of gga-miR-140-5p as a candidate miRNA involved in IMF deposition based on the result of miRNA sequencing of intramuscular adipocyte differentiation model. Therefore, it can be concluded that miRNAs have different expressions in various tissues, ages, and developmental stages.

##### Gga-miR-18b-3p

Mir-sequencing and functional enrichment analysis of the intramuscular adipocyte differentiation model revealed that gga-miR-18b-3p is considered as an inhibitor of intramuscular adipocyte differentiation by negatively targeting 3′-UTR of acyl-CoA thioesterase 13 (ACOT13) gene. Both miR-18b-3p and ACOT13 are differentially expressed between IMF-pre-adipocytes and IMF-adipocytes. Therefore, the miR-18b-3p is involved in the process of IMF adipogenesis through negatively targeting ACOT13. It should be emphasized that the ACOT gene family has a significant role in fatty acid biosynthesis and is greatly expressed in oxidative tissues, such as the liver and adipose tissue [57].

##### Gga-miR-let-7

Based on the literature, the miR-let-7 family involves in both muscle development and fat deposition in an animal [15]. Among let-7 family members, let-7b plays a significant role in chicken skeletal muscle growth and fat deposition by targeting growth hormone receptor (GHR) expression [68].

## 6. Main Signaling Pathways Involved in Chicken Fat Metabolism

**Figure 4:**
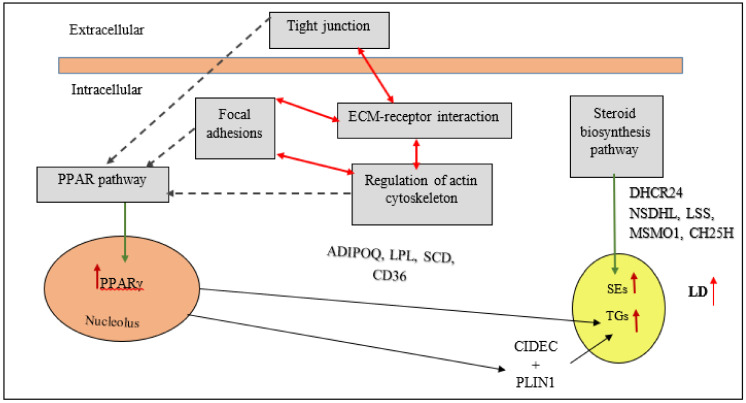
The potential network of signaling pathways contributing to the chicken lipid metabolism. The above-mentioned pathways (gray boxes) might be the crucial pathways for lipid deposition in chicken breast muscle. Activation of PPARγ in the PPAR signaling pathway leads to upregulation of lipogenic genes, including ADIPOQ, LPL, SCD, or CD36, to promote TG synthesis. Moreover, PPARγ may promote the interaction of PLIN1with CIDEC to accelerate LD formation. Meanwhile, upregulation of cholesterol synthesis genes, such as DHCR24 and NSDHL, in the steroid biosynthesis pathway may upgrade the steroid ester synthesis. Ses, Sterol esters; TGs, Triglycerides; LD, lipid droplet; PLIN, Perilipins; PPARγ, peroxisome proliferator-activated receptor γ; ADIPOQ, adiponectin; LPL, lipoprotein lipase; SCD, stearoyl-CoA desaturase; CD36, cluster of differentiation 36; DHCR24, 24-dehydrocholesterol reductase; NSDHL, NAD (P) dependent steroid dehydrogenase-like; LSS, Lanosterol synthase; MSMO1, Methylsterol monooxygenase 1; CH25H, Cholesterol 25-hydroxylase. Black, red, and green arrows indicate possible regulatory relationship, bidirectional regulatory relationship, and reported regulatory relationship, respectively. Figure 4 was adapted from [23] with permission.

In different organisms, miRNAs, through complicated interactions with their target genes, participate in the regulation of various biological processes, such as abdominal and intramuscular adipose tissue development [18]. In chicken, KEGG pathway analysis of genes targeted with differentially expressed miRNAs in the intramuscular adipocyte differentiation model indicated that these genes are significantly enriched in the pathways involved in fatty acid metabolism, including the PPAR signaling pathway, fatty acid metabolism, and fatty acid degradation [57]. Furthermore, the miRNA-mRNA interaction network analysis involved in abdominal fat development in Gushi chicken showed that there were two signaling pathways related to adipogenesis and lipid metabolism, namely, PPAR and adipocytokine signaling pathways. These two signaling pathways were connected to each other by the key gene named adiponectin (ADIPOQ), which was targeted by several miRNAs, including let-7 miRNAs family (let-7a-5p, let-7b, let-7c-5p, etc.), miR-155, miR-125b-3p, miR-218-5p, miR-30a-3p, and miR-30e-3p [27]. Moreover, PPAR signaling and steroid biosynthesis pathways are the significant contributors of lipid deposition in chicken pectoralis muscle tissue [6,18]. Likewise, the expression of cholesterol synthesis-related genes, such as 24-dehydrocholesterol reductase (DHCR24) and NAD(P) dependent steroid dehydrogenase-like (NSDHL) in steroid biosynthesis pathway may promote steroid ester synthesis, which along with PPAR signaling pathway may contribute to lipid metabolism augmentation in chicken muscle tissue [23] (Figure 2). On the other hand, it has been indicated that cell junction pathways, such as focal adhesions and EMC-receptor interaction, as well as the PPAR signaling pathway, might regulate fat deposition and accumulation during chicken development. Therefore, it can be inferred that chicken fat deposition is regulated and mediated not only by genes related to lipid metabolism and PPAR signaling pathway, but also by other pathways involved in cell junction intending to maintain the integrity of tissues, as well as signal transduction [6] (Figure 4).

The genes involve in the PPAR signaling pathway, including PPARγ, FABP3, FABP4, RXRA, LPL, acyl-CoA synthetase family member 3 (ACSF3), acyl-CoA synthetase short-chain family member 2 (ACSS2), CD36, carnitine palmitoyltransferase 2 (CPT2), cytochrome P450, family 27, subfamily A, and polypeptide 1 (CYP27A1), are functional genes in the chicken lipid metabolism [6]. Among these genes, the PPARγ, which is the key transcriptional factor in adipocyte differentiation, is targeted with miR-200b-3p in the interaction network associated with the PPAR signaling pathway [27] (Figure 2). In addition, miRNAs participating in the lipid metabolism, including miR-499-5p, miR-32-5p, miR-200a-5p, miR-31-5p, miR-34c-5p, miR-449a, miR-146a-5p, and miR-221-3p, are able to connect different signaling pathways, including fatty acid metabolism, degradation, and biosynthesis, unsaturated fatty acid biosynthesis, and α-linolenic acid metabolism by targeting key genes, such as ACSL, bubblegum family member 2 (ACSBG2), acyl-CoA oxidase 1 (ACOX1), and FADS2 [27].

The gene ontology (GO) enrichment analysis revealed that significant differentially expressed miRNAs in the chicken liver targeted various genes which mainly involve in the pathways related to the lipid-related metabolic processes, such as steroid biosynthesis, glycerophospholipid metabolism, biosynthesis of unsaturated fatty acids, and the PPAR signaling pathway [18]. Furthermore, during chicken adipogenesis, several signaling pathways, such as Wnt and Hedgehog signaling pathways, are involved in the mesenchymal stem cell’s transformation to an adipogenic lineage. In fact, the Wnt signaling family members by inhibiting the expression of PPARγ and C/EBPα lead to repression in the early stages of adipogenesis. Similarly, the Hedgehog signaling pathway also leads to adipogenesis suppression by activation of anti-adipogenic transcription factors, such as the GATA binding protein 2 (GATA2) [31]. There are several studies, investigating the main signaling pathways involved in lipid metabolism in chicken with divergent fat content, ages, and breeds. As an example, the RNA deep sequencing of liver from chicken with divergent unsaturated fatty acids content in their breast muscle revealed that the most dominant enriched pathways which have a significant role in unsaturated fatty acid metabolism were ECM-receptor interaction, focal adhesion, peroxisome, Wnt, and TGF-β signaling pathways [33]. On the other hand, the RNA sequencing of pectoralis muscle from broiler with divergent TG content revealed that key pathways controlling divergent lipid deposition and metabolism in chickens with high and low TG content were steroid biosynthesis (participating in cholesterol synthesis), PPAR signaling pathway, and cell junction-related pathways (focal adhesion, cell adhesion, tight junction, ECM-receptor interaction, regulation of actin cytoskeleton) [23] (Figure 4). The differential miRNA expression profiling in the liver of 20- and 30-week old hen and their predicted target genes revealed that some of these genes were significantly involved in the processes related to lipid metabolisms, such as steroid biosynthesis, glycerophospholipid metabolism, unsaturated fatty acid biosynthesis, and the PPAR signaling pathway [18]. The expression analysis of miRNAs in abdominal adipose tissue of Gushi chicken during 6, 14, 22, and 30 weeks of ages revealed that the significant differentially expressed miRNA primarily involved in the modulating of different biological processes, including fatty acid metabolism, lipid metabolism, unsaturated fatty acid metabolism, and fat cell differentiation. These biological processes were significantly different at various chicken abdominal fat developmental stages; specifically during 14 to 22 weeks of age as the most important stage for abdominal fat development in chicken. [69]. Overall, it is well documented that there is a complex regulatory network constructed by the interactions between miRNAs and genes and between pathways that regulates fat metabolism and abdominal adipose tissue development [27]. Based on the aforementioned studies, the most probable key pathways for lipid metabolism and deposition in the chicken are the PPAR signaling pathway (main and most important pathway in the regulation of lipid metabolism among muscle, liver, and adipose tissue), cholesterol synthesis, calcium signaling and cell-junction related pathways (ECM-receptor interaction, focal adhesion), Wnt signaling pathway, and insulin signaling pathway.

## 7. Hormonal Regulation of Fat Metabolism in Chicken

Since there is an inverse correlation between various miRNA and their target genes in chicken hepatocytes, it is revealed that certain miRNAs and their predicted target genes are controlled by several hormones, such as growth hormone (GH), in the chicken liver [30]. GH is secreted by the anterior pituitary gland, which can be the regulator of animal growth and development, as well as protein, carbohydrates, and lipid metabolism. The RNA sequencing of GH-treated and untreated chicken hepatocytes indicated that GH can regulate different metabolism in the liver, particularly lipid metabolism. In fact, GH, by regulating the expression of miRNA targeting the genes involved in lipid metabolism, can modulate the lipid metabolism in the chicken liver. For instance, two important genes involved in lipid metabolism, which are regulated by GH, are FABP1and phosphatidic acid phosphatase type 2B (PPAP2B). Subsequently, RNA sequencing of primary hepatocytes in female chickens was indicated that some miRNAs upregulated with GH, including gga-miR-223, gga-miR-193a, gga-miR-190, and miR-15b. While gga-miR-451 was downregulated by GH and miR-122 was not involved in GH regulation of liver metabolism. In which, miR-15b by targeting the genes related to lipid metabolism, including ACSL3, endothelial lipase (LIPG), phospholipase C, delta 1 (PLCD1), PPAP2B, and steroidogenic acute regulatory protein (STAR), is a very important miRNA mediating the effect of GH on lipid metabolism in the chicken liver [13]. Another secreted hormone in chicken is insulin which may affect the expression of genes related to glucose and lipid metabolism, therefore affecting fat accumulation in chickens [9]. Studies have shown that insulin can stimulate the expression of the genes to encode important enzymes in lipogenesis, including MD, SCD, FASN, and LPL [20] (Figure 1 and Figure 2). On the other hand, published data have revealed that sex hormones are vital for adipogenesis regulation and fat deposition in chickens. Among sex hormones, estrogen can regulate important genes participating in different processes, such as fat synthesis, fat transportation, and oxidation [57]. Likewise, estrogen by downregulating the expression of miR-218-5p in the liver of egg-laying hen can increase the expression of its target gene, ELOVL5, which leads to enhanced hepatic n-3 and n-6 LCPUFA synthesis [44]. Moreover, the increased estrogen level in the plasma of egg-laying hens during the egg-laying period led to a significant increase in synthesis and expression of ApoB, as a gene with a vital role in the secretion of triglyceride-rich lipoproteins; while miR-101-2-5p can negatively regulate the expression of ApoB in the chicken liver [64]. Overall, hormonal control has been considered an important factor in the regulation of the hepatic lipogenesis rate in chickens [20]. Thus, hormonal background, as well as genetic selection, are important determinates of divergent hepatic lipogenesis rate in different chicken breeds [21].

## 8. Conclusions

Since diminishing accretion of adipose tissue is a novel challenge of the poultry industry to increase the profitability of production and meat quality [70], this review was designed to elucidate the complexity of lipid metabolism and deposition in chicken, which is orchestrated by a series of regulatory factors at the transcriptional and post-transcriptional regulatory level; including (1) lipid-related genes, comprising of genes involved in chicken lipogenesis: ACLY, ACACA, FASN, SCD, ELOVLs, and FADS2; genes involved in TG synthesis: Acyl-CoA synthetase, GPAT, AGPAT2, DGAT2 and MTTP; and genes involved in chicken adipogenesis and fat deposition: LPL, FADS4, FADS5. (2) Transcription factors, such as PPARγ and C/EPBα, play a dominant role in chicken adipogenesis by activating the expression of adipogenic-specific genes, such as FABP4, SREBP1, and FASN, therefore promoting the storage of TG and lipid accumulation. (3) Different hormones, including growth hormone, insulin, and estrogen. (4) Subsequently, it is revealed that miRNAs have significant effects on the regulation of lipid metabolism and deposition by targeting lipid-related genes and transcription factors in chicken. The most abundant expressed miRNAs in chicken are miR-122, miR-148a-3p, miR-let-7 and miR-30 families, miR-146b-5p, miR-19-3p, miR-10, miR-33, miR-17-3p. It should be taken into consideration that various miRNAs have different expression rates in different chicken tissues (liver, adipose tissue, and breast muscle), ages, developmental stages, breeds, and gender. Therefore, more precise and classified studies are needed to investigate the expression of significant miRNAs in various chicken breeds at different developmental stages and tissues. Finally, outcomes from this review can create new insight into the transcriptional and post-transcriptional regulatory mechanism of lipid metabolism and fat deposition in chicken, which can lead to improvement of the meat quality in chicken, as well as facilitating identification of functional miRNAs as the genetic marker to enhance quality and nutritional value of chicken meat.

## Figures and Tables

**Table 1:** Summary of the significantly expressed miRNAs associating with lipogenesis in the chicken liver.

miRNA	Function	References
Gga-miR-122	Liver-specific miRNA Hepatic fatty acid oxidation, fatty acid, and cholesterol synthesis regulator	[18,47,61]
nc-miR-5 (novel)	Target C/REB1 and PBP/PPARBP Involves in lipogenesis and liver growth	[47]
nc-miR-33 (novel)	Regulate FASN expression in embryonic chicken liver Involves in lipogenesis and liver growth	
Gga-miR-33	Involve in lipid metabolism and energy homeostasis by negatively regulating the expression of CROT, HADHB, and FTO genes in the chicken liver	[3,60]
Gga-miR-22-3p	Targeting ACSL5 and ELOVL6 as important regulators of chicken hepatic lipid synthesis	[18]
Gga-miR-33a	Regulators of cholesterol homeostasis and fatty acid metabolism by targeting SREBP1, which is the regulator of genes involved in lipogenesis	
Gga-miR-33b		
Gga-miR-146b-5p	Regulate lipid metabolism in chicken liver Adipogenesis regulator	
Gga-miR-24-3b	Its overexpression can promote hepatic liver accumulation	
Gga-miR-148a-5p	Regulate hepatic lipid metabolism	
Gga-miR-21-5p		
Gga-let-7F-5p		
Gga-miR-26a-5p		
MiR-126-5p		
Gga-miR-30d		
Gga-miR-10a-5p		
Gga-miR-128-3p	Targeting genes enriched in the glycerophospholipid metabolism pathway	
Gga-miR-15b	Mediating the effect of GH on lipid metabolism in chicken liver	[13]
Gga-miR-218-5p	Estrogen negatively regulates the expression of these miRNAs, which leads to an increase I the expression of ELOVL5 and biosynthesis of n-3 and n-6 LCPUFA	[44]
Gga-miR-19-3p		
Gga-miR-30-5p		
Gga-miR-101-2-5p	Involve in lipid metabolism by negatively regulate the expression of ApoB gen in the liver of chickens	[64]
Gga-34a-5p	Increasing the intracellular levels of triglycerides, as well as total cholesterol in hepatocyte by inhibiting the translation of ACSL1 in chicken livers	[46]

**Table 2:** Summary of the significantly expressed miRNAs in chicken abdominal fat.

miRNA	Function	References
Gga-let-7a	Highly abundant differentially expressed miRNAs in the chicken abdominal fat	[41,65]
Gga-let-7j		
Gga-let-7b		
Gga-let-7f		
Gga-let-7c		
Gga-let-7k		
Gga-miR-148a-3p	Abundant miRNA in abdominal fat of lean broiler lines Promote adipogenesis by targeting WNT1	[27,65]
Gga-miR-146c	Involve in chicken preadipocyte development and metabolism	[65]
Gga-miR-10a		
Gga-miR-33		
Gga-miR-222		
Gga-miR-15a		
Gga-miR-22		
Gga-miR-206		
Gga-miR-1a		
Gga-miR-29b		
Gga-miR-9		
Gga-miR-32		
Gga-miR-429	Regulate adipogenesis	
Gga-miR-200b		
Gga-miR-451		
Gga-miR-142-5p		
Gga-miR-200a		
Gga-miR-218		
Gga-miR-454 Gga-miR-30a-5p [27]		
Gga-miR-122-5p	Adipose-related miRNA Involve in growth and development of adipose tissue by targeting PPARγ Its abundance affects the PPAR signaling pathway and adipocyte differentiation	[67]
Gga-miR-19b-3p	Promoting preadipocyte proliferation and adipocyte differentiation by downregulation of ACSL1	[14]
Gga-miR-19a-3p	Involve in regulating the accumulation of abdominal fat in chicken	
Gga-miR-17-5p		
Gga-miR-26a		
Gga-miR-103-3p		
Gga-miR-92-3p		
Gga-miR-34	Have key roles in: Lipid metabolism Adipocyte proliferation and differentiation Cell junctions during abdominal adipose tissue development in chicken	[27]
Gga-miR-199		
Gga-miR-8		
Gga-miR-146b-5p		
Gga-miR-17-3p	Promote adipocyte differentiation by targeting fatty acyl desaturase gene	
Gga-miR-215-5p	Differentially expressed miRNAs in different stages of abdominal adipose tissue development	
Gga-miR-499-5p		
Gga-miR-135a	MiRNAs with developmental stage-specificity Regulate adipocyte differentiation	
Gga-miR-21	Inhibit chicken preadipocyte proliferation by downregulating KLF5 Abundant miRNA in abdominal fat of fat broiler lines Regulate adipogenesis	[9,27,53,65]
Gga-miR-101-3p	Regulate adipogenesis Involve in chicken preadipocyte development and metabolism	[27,65]
Gga-miR-31	MiRNA with developmental stage-specificity Regulate adipocyte differentiation Involve in chicken preadipocyte development and metabolism	[27,65]
Gga-miR-30d Gga-miR-27-3p	Regulate adipogenesis Involve in regulating the accumulation of abdominal fat in chicken	[14,27]

**Table 3:** Summary of the significantly expressed miRNAs associated with intramuscular adipogenesis in chicken.

miRNA	Function	References
Gga-Let-7b	Having a significant role in chicken skeletal muscle growth and fat deposition by mediating GHR expression Regulates adipogenesis	[15,68]
Gga-miR-30a	Key regulators of adipogenesis	[15]
Gga-miR-30d		
Gga-miR-30C	Promote adipogenic differentiation	
Gga-miR-17-5p	Regulate: Fat deposition Adipogenic differentiation	
Gga-miR-20a-5p Gga-miR-10b-5p		
Gga-miR-148a-3p	Promotes fat synthesis by inhibition of Wnt 1 gene expression Regulates adipogenic differentiation of mesenchymal stem cells	
Gga-miR-122-5p	Regulates lipid metabolism	
Gga-miR-31-5p	Regulate adipogenic differentiation	
Gga-miR-27b-3P Gga-miR-24-3p		
Gga-miR-103-3p Gga-miR-146b-5p	Promotes adipogenesis and fat deposition	
Gga-miR-22-5p	Inhibits adipogenic differentiation of adipose tissue	
Gga-miR-140-5p	Targeting RXRG gene and promote intramuscular adipocyte differentiation	[7]
Gga-miR-19b-3p	Regulate preadipogenic differentiation (Differentially expressed miRNAs between juvenile and late-laying period hen)	
Gga-miR-425-5p		
Gga-miR-27a		
Gga-miR-146-5p		
Gga-miR-223	Negative regulator of chicken intramuscular preadipocyte differentiation by targeting GPAM gene	[7,52]
Gga-miR-18b-3p	Regulates intramuscular adipocyte differentiation	[57]

## Data Availability

No new data were created or analyzed in this study. Data sharing is not applicable to this article.
